# Self‐reported cognitive outcomes among adolescent and young adult patients with noncentral nervous system cancers

**DOI:** 10.1002/pon.5456

**Published:** 2020-07-09

**Authors:** Chia Jie Tan, Jaclyn Jia Jun Mah, Wei Lin Goh, Eileen Poon, Mohamad Farid Harunal Rashid, Alexandre Chan

**Affiliations:** ^1^ Department of Pharmacy National University of Singapore Singapore Singapore; ^2^ Department of Pharmacy National Cancer Centre Singapore Singapore Singapore; ^3^ Division of Medical Oncology National Cancer Centre Singapore Singapore Singapore; ^4^ Duke‐NUS Medical School Singapore Singapore; ^5^ Department of Clinical Pharmacy Practice University of California Irvine California USA

**Keywords:** adolescent, cancer, cognitive impairment, distress thermometer, FACT‐Cog, oncology, psycho‐oncology, young adult

## Abstract

**Objective:**

Cancer‐related cognitive impairment (CRCI) among adolescent and young adult (AYA) cancer patients with noncentral nervous system (CNS) cancers has not been well studied. In this study, we aimed to describe CRCI‐associated trends and characteristics among AYA cancer patients.

**Methods:**

In a longitudinal cohort of AYA cancer patients without CNS disease, CRCI was evaluated over 1 year using the Functional Assessment of Cancer Therapy‐Cognitive Function Instrument, a self‐reported cognitive outcome measure. CRCI prevalence was quantified using the previously established minimal clinically important difference. CRCI‐associated longitudinal trends and factors were evaluated with mixed‐effects model analysis.

**Results:**

Ninety‐one patients (mean age = 28.4 ± 6.7 years) were included. Approximately one‐third (34.1%) experienced CRCI at least once during the study follow‐up. Female gender (*P* = .02), Indian ethnicity (*P* < .01), current smokers (*P* < .01), anxiety/depressive symptoms (*P* < .01) and fatigue (*P* < .01) were found to be associated with poorer cognitive function among AYAs.

**Conclusions:**

Although AYA cancer patients were relatively young and without CNS disease involvement, a significant proportion of them experienced clinically important decline in cognitive function. With improved understanding of this subject, effective strategies can be formulated to promote awareness of CRCI and mitigate its negative effects among AYA cancer patients.

## 1 INTRODUCTION

Cancer‐related cognitive impairment (CRCI) is one of the complications that plague cancer patients from the point of diagnosis to beyond disease remission, even in the absence of organic causes such as central nervous system (CNS) tumors. The International Cancer and Cognition Taskforce (ICCTF) defines CRCI as a decline in memory, attention, concentration, and executive function among cancer patients.^1^ Extensive research has been performed in order to evaluate CRCI in single tumor type cohorts, for example, breast and colorectal cancer patients. However, the mean ages of the study subjects in these studies are generally much higher than the adolescent and young adult (AYA) age range,^2^, ^3^ which encompasses cancer patients between the ages of 15 and 39 years old, as defined by the Institute of Medicine.^4^ Even among cancers that are common within the AYA age range, such as Hodgkin's lymphoma^5^ and testicular cancer,^6^ representation of AYA patients is often low, and subgroup analysis of AYA patients has not been presented in the literature. Age‐specific CRCI studies have also been limited thus far to survivors of childhood cancers^7^ and the elderly,^8^ indicating a lack of robust studies quantifying the prevalence and characterizing CRCI trends in AYA patients diagnosed with malignancies.

Preliminary data suggest that AYAs are encumbered by CRCI at work or in school. The AYA Health Outcomes and Patient Experience study reports that up to 53% of working cancer survivors within this age range face problems with memory or paying attention at work or school.^9^ In another cross‐sectional study, AYAs who have undergone chemotherapy were also approximately 2 to 4 times more likely to experience difficulties with mental tasks while working than those who did not receive chemotherapy.^10^ These issues are detrimental to AYAs, who are in a critical junction in life with many developmental milestones to achieve, including completing their education and embarking on a career.^11^ Furthermore, the productivity loss associated with AYAs is disproportionately large since they serve as the backbone of the economy.^12^ For these reasons, unaddressed CRCI can potentially lead to devastating consequences for both AYA cancer patients and society at large. It is therefore imperative to comprehensively evaluate and understand CRCI among AYA cancer patients.

We have previously conducted a longitudinal assessment of symptom burden and distress in a cohort of AYA cancer patients.^13^ In this article, the self‐reported cognitive function of patients with nonCNS malignancies will be reported. Furthermore, CRCI‐associated characteristics will be identified. Our findings will be important to demonstrate if CRCI is prevalent in the AYA population.

## 2 METHODS

### 2.1 Study design

This was a secondary analysis of self‐reported cognitive outcomes collected from a longitudinal cohort study at the National Cancer Centre Singapore. The study was conducted from September 2015 to October 2018. Ethics approval was obtained from SingHealth Centralized Institutional Review Board (2015/2011).

### 2.2 Study population

Recruitment was carried out at outpatient oncology clinics. Patients who were newly referred to a medical oncologist and fulfilled the eligibility criteria were referred to the study team by their physicians. The inclusion criteria were: (a) between 15 and 39 years old and (b) an official cancer diagnosis. Those who were unable to provide informed consent, had serious comorbid illnesses (such as neuropsychiatric conditions, which would severely affect quality of life), or would not have any scheduled visits subsequently were ineligible. For the purpose of this analysis, patients with organic causes of cognitive decline, such as primary and secondary CNS tumors, were excluded. All participants provided written informed consent prior to joining the study.

### 2.3 Data collection

Participants were assessed at four time points over 1 year: (a) baseline at recruitment (T1); (b) 1 ± 0.5 month later (T2); (c) 6 ± 2 months later (T3); and (d) 12 ± 2 months later (T4). The three follow‐up time points were intended to correspond to the active treatment period, when treatment had just completed and during the survivorship phase. At baseline, participants provided demographic information via a self‐administered questionnaire. Information included age, gender, ethnicity, education level, smoking status, and alcohol consumption. Medical history and clinical parameters, such as diagnosis, disease staging, Eastern Cooperative Oncology Group (ECOG) performance status, and treatment details were extracted from electronic medical records. At each time point, participants were also requested to complete a series of self‐administered patient‐reported outcome (PRO) questionnaires described below.

#### 2.3.1 Self‐reported cognitive function

The Functional Assessment of Cancer Therapy‐Cognitive Function (FACT‐Cog) version 3 is a 37‐item self‐reported questionnaire designed to assess CRCI.^14^ FACT‐Cog has been widely used and validated in our local cancer population^15^ and consists of 4 subscales, which are (a) perceived cognitive impairment (PCI), (b) comments from others (OTH), (c) perceived cognitive abilities (PCA), and (d) impact on quality of life (QOL). Scores of negatively worded items were reversed such that higher values indicated better self‐perceived cognitive function. The total score was obtained by summing scores from all subscales. A decrease of more than 10.6 in total score has previously been established to indicate clinically important deterioration among Asian breast cancer patients.^16^ The questionnaire developer has also recommended presenting data from individual subscales and using the PCI score as one of the primary measures,^17^ where a score of less than 60 has been used to identify CRCI cases.^18^ As suggested by the questionnaire developer, four items (MT1, MT2, PMT1, and PMT2) were only included in the score calculation after they were evaluated to fit with the scales, as indicated by Cronbach's alpha values of >.9 and item‐to‐scale correlation >.7.

#### 2.3.2 Distress and symptom burden

Distress thermometer (DT) is a brief screening tool that measures distress. A problem checklist is also provided for respondents to identify items that have posed difficulties for them in the past week, including memory or concentration. The Rotterdam Symptom Checklist (RSCL) is used to assess the symptom burden of cancer patients. In this study, RSCL was also used to measure anxiety/depressive symptoms^19^ and significant fatigue.^20^ Details of both tools are provided in Supporting Information S1.

### 2.4 Statistical analysis

Statistical analyses were conducted with STATA Version 15 (StataCorp, 2017). Descriptive statistics were used to describe the demographic and clinical characteristics of the study population and to illustrate self‐perceived CRCI trends as reported by the participants. Differences in continuous and categorical variables between participants with and without complete data were compared using two‐tailed Student's *t*‐tests and the Chi‐square test, respectively. Longitudinal trends and factors associated with cognitive function were assessed using linear mixed‐effects models with the assessment time point (modeled as a categorical variable) incorporated as a fixed effect and the intercept varied as a random effect for each individual participant. In order to assess the influence of treatment modality on cognitive function, the exposure of each participant to chemotherapy, surgery, or radiotherapy at each time point was forced into the model as a fixed effect. Other demographic and clinical parameters were incorporated into the model in a stepwise manner with Akaike Information Criterion (AIC) and Bayesian Information Criterion (BIC) used to guide model building. Lower AIC and BIC values indicated model stability. A sensitivity analysis was conducted using the PCI score as the dependent variable in the final model. In order to compare the concordance between DT and FACT‐Cog when determining CRCI, percentage agreement and Cohen's kappa coefficient were calculated. For all statistical tests performed, a *P*‐value of <.05 was considered statistically significant. Adjustment for multiple comparisons was not carried out due to the exploratory nature of our study.

## 3 RESULTS

### 3.1 Demographic and clinical characteristics

A total of 91 cancer patients with a mean age of 28.4 years old (range: 16‐39 years old) completed cognitive evaluation at baseline (Supporting Information S2). At recruitment, the median time after diagnosis was 0.8 months (range: 0‐4.5 months), with sarcomas (42.9%) and lymphomas (33.0%) being the two most common malignancies. Other clinical and demographic characteristics are described in Table 1. A summary of the patients' distress level and symptom burden can be found in Supporting Information S1.

**TABLE 1:** Participant characteristics

Characteristics	N = 91
Age in years, mean (SD)	28.4 (6.7)
Gender, n (%)	
Male	49 (53.8)
Female	42 (46.2)
Race, n (%)	
Chinese	64 (70.3)
Malay	8 (8.8)
Indian	6 (6.6)
Others^a^	13 (14.3)
Highest education level completed, n (%)	
Primary/secondary education	10 (11.0)
Pre‐university^b^	21 (23.1)
Bachelor's degree	29 (31.9)
Postgraduate degree	29 (21.9)
Unreported	2 (2.2)
Smoking status, n (%)	
No history of smoking	62 (68.1)
Currently smoking	12 (13.2)
Previously smoking	14 (15.4)
Unreported	3 (3.3)
Alcohol, n (%)	
No	56 (61.5)
Yes	32 (35.2)
Unreported	3 (3.3)
Time after diagnosis in months, median (IQR)	0.8 (0‐1.8)
Cancer type, n (%)	
Sarcoma	39 (42.9)
Lymphoma	30 (33.0)
Germ cell tumor	12 (13.2)
Melanoma	8 (8.8)
Pancreatic neoplasm	1 (1.1)
Nasopharyngeal neoplasm	1 (1.1)
ECOG at diagnosis, n (%)	
0	59 (64.8)
1	29 (31.9)
2	3 (3.3)
Cancer stage, n (%)	
I	27 (29.7)
II	18 (19.8)
III	11 (12.1)
IV	23 (25.3)
Not applicable	12 (13.2)
Disease nature	
New diagnosis	78 (85.7)
Relapsed/refractory disease	13 (14.3)
Treatment modality	
Chemotherapy	52 (57.1)
Surgery	44 (48.4)
Radiotherapy	25 (27.5)

^a^ Burmese, Filipino, and Arabian.

^b^ Refers to colleges which lead to Singapore‐Cambridge GCE A‐levels and vocational diplomas.

Eighty‐two (90.1%) participants had complete data for at least one follow‐up time point while 47 participants (51.6%) had complete data for all study time points. The demographic characteristics of participants who had complete data at each study time point were largely similar to those with missing data. Of note, no significant difference between baseline self‐reported cognitive function was observed between participants who had complete and incomplete data at all study time points (Supporting Information S3).

### 3.2 Subjective CRCI

**FIGURE 1:**
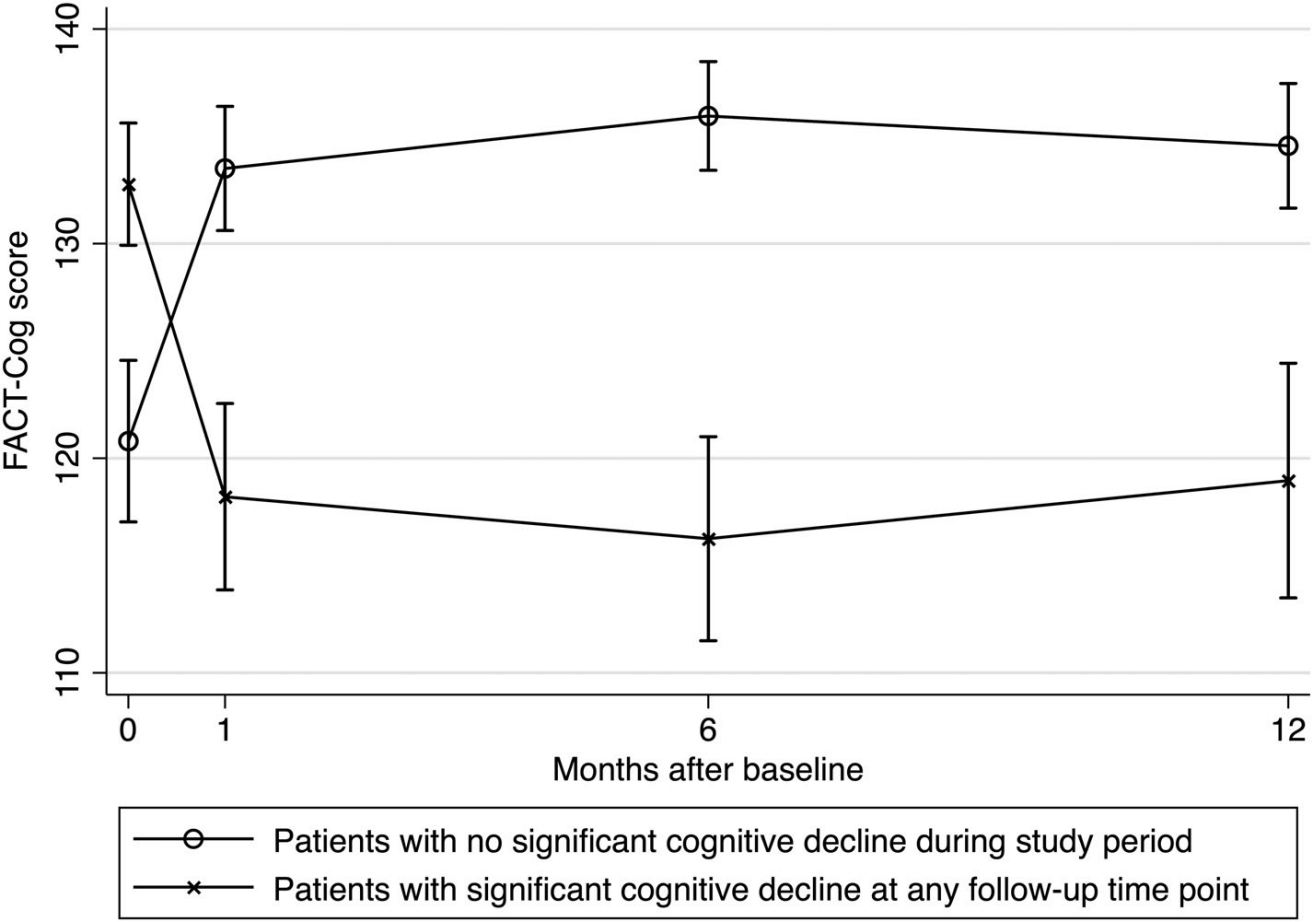
Longitudinal trends of FACT‐Cog scores. Patients who exhibited significant cognitive decline at any follow‐up time point were found to have higher baseline FACT‐Cog total scores, which deteriorated subsequently during the study period

The mean change in FACT‐Cog total and subscale scores were small in magnitude at all follow‐up time points and mostly indicated improvement of cognitive function over time (Table 2). Longitudinal analysis did not reveal significant changes in the mean scores over time, except for the QOL subscale (Table 2). However, based on the minimal clinically important difference (MCID) of FACT‐Cog, 22.5%, 25.7%, and 20.7% of participants exhibited self‐reported CRCI at T2, T3, and T4, respectively. Overall, 34.1% of participants reported CRCI at least once during the study period. Patients who exhibited CRCI at any time point were found to have higher baseline FACT‐Cog scores, indicating better self‐reported cognitive function at recruitment (138.6 vs 128.3; *P* = .04; Figure 1). Self‐reported cognitive function in this group of patients noticeably deteriorated at subsequent time points with a mean reduction of 14.2, 16.6, and 13.3 points at T2, T3, and T4, respectively.

**TABLE 2:** Longitudinal trends in FACT‐Cog total and subscale scores

	Total score, mean (SD)	Subscale scores, mean (SD)			
		PCI	OTH	PCA	QOL
*Baseline, T1 (N = 91)*					
Raw score	124.9 (26.0)	70.8 (14.2)	15.2 (2.2)	28.8 (8.8)	10.1 (5.6)
*1 month after baseline, T2 (N = 71)*					
Raw score	127.7 (21.7)	72.5 (10.8)	15.4 (1.4)	27.8 (10.0)	12.0 (4.6)
Change (T2‐T1)	1.3 (19.1)	0.7 (8.8)	0.0 (2.0)	−1.3 (9.6)	1.9 (5.2)
*6 months after baseline, T3 (N = 70)*					
Raw score	128.1 (22.4)	72.8 (11.3)	15.3 (1.7)	26.6 (11.3)	13.3 (3.7)
Change (T3‐T1)	1.7 (24.3)	1.2 (12.9)	−0.1 (2.4)	−2.2 (11.6)	2.8 (5.9)
*12 months after baseline, T4 (N = 58)*					
Raw score	128.1 (22.7)	71.8 (12.2)	15.3 (2.0)	27.7 (10.1)	13.1 (4.0)
Change (T4‐T1)	0.7 (21.5)	−0.1 (12.4)	−0.4 (2.2)	−2.0 (9.6)	3.1 (5.2)
*P* value^a^	.79	.55	.91	.38	<.01

Abbreviations: PCI (perceived cognitive impairment), OTH (comments from others), PCA (perceived cognitive abilities), QOL (impact on quality of life).

^a^ Longitudinal change in FACT‐Cog score evaluated with mixed‐effects model analysis.

Similar to FACT‐Cog, a proportion of participants also indicated problems with memory or concentration on the DT problem checklist at each time point (Supporting Information S4). Percentage agreement between self‐perceived CRCI assessed using total FACT‐Cog score and reports of memory or concentration as a concern on the DT was approximately 74% to 75% across all follow‐up time points. Cohen's kappa values suggested that concordance was poor (Supporting Information S4). However, greater concordance was observed when comparing CRCI assessed using the PCI subscale score against the DT problem checklist, with higher Cohen's kappa scores in the range of 0.4 to 0.7 (Supporting Information S4).

### 3.3 Factors associated with self‐reported cognitive function

After controlling for time, the mixed‐effects model analysis showed that none of the treatment modalities (chemotherapy, surgery, or radiotherapy) were significantly associated with self‐reported cognitive function. Based on model diagnostics (Supporting Information S5), gender, ethnicity, and smoking status were included in the final model. In our analysis, female gender (*P* = .02), Indian descent (*P* < .01), and smoking (*P* < .01) were associated with a higher likelihood of reporting cognitive‐associated complaints. Psychosocial factors, such as anxiety/depressive symptoms (*P* < .01) and fatigue (*P* < .01), both measured using the RSCL, were also associated with worse self‐perceived cognitive function (Table 3). Age and education level were found to be nonsignificant predictors of self‐perceived cognitive function and were excluded from the final model (Supporting Information S5). The statistical significance of associations remained unchanged when the PCI subscale score was used as the outcome measure to represent subjective cognitive function (Supporting Information S6).

**TABLE 3:** Factors associated with cognitive function (measured based on total FACT‐Cog score) among AYA cancer patients (N = 91)

	Beta coefficient	95% CI	*P*‐value
Chemotherapy			.62
No	Reference		
Yes	1.70	−5.0 to 8.4	
Surgery			.86
No	Reference		
Yes	−0.60	−7.4 to 6.2	
Radiotherapy			.15
No	Reference		
Yes	−5.5	−13.0 to 2.0	
Gender			.02
Male	Reference		
Female	−7.9	−14.5 to −1.3	
Ethnicity			<.01
Chinese	Reference		
Malay	2.1	−10.3 to 14.4	
Indian	−33.6	−47.2 to −19.9	
Others^a^	−0.17	−9.8 to 9.4	
Anxiety/depressive symptoms^b^			<.01
No	Reference		
Yes	−16.6	−23.4 to −9.8	
Fatigue^c^			<.01
No	Reference		
Yes	−12.1	−16.8 to −7.4	
Smoking habit			<.01
No history of smoking	Reference		
Currently smoking	−19.3	−30.1 to −8.5	
Previously smoking	−8.5	−17.6 to 0.6	

^a^ Burmese, Filipino, and Arabian.

^b^ RSCL psychological scale score >16.

^c^ RSCL fatigue item score >2.

## 4 DISCUSSION

In this longitudinal study, one‐third of the participants experienced self‐reported CRCI at least once within a span of 12 months, despite their relatively young age and absence of CNS‐associated cancer. Compared to previous studies investigating subjective CRCI, the proportion of patients with subjective CRCI in our study was lower than that observed among breast cancer patients receiving chemotherapy (20%‐50%)^21^, ^22^, ^23^ but higher than patients who did not undergo chemotherapy (10%‐15%).^21^ In contrast to other studies, the mean deterioration in FACT‐Cog total and subscale scores observed in our study were smaller in magnitude. Among breast cancer patients, mean changes of −10.4 and −6.5 in FACT‐Cog total and PCI subscale scores, respectively, were reported at completion of chemotherapy.^21^ Similarly, colorectal cancer patients reported a decrease of 9.4 and 5.7 in FACT‐Cog total scores at 6 and 12 months after initiation of chemotherapy.^24^ These observations suggest that CRCI is a less prominent problem in the AYA cancer population as a whole.

Nevertheless, in view of the significant proportion of AYA cancer patients who experienced clinically important CRCI, it is vital to identify individuals who are susceptible to CRCI. This is because cancer‐related complications have been suggested to reduce the performance of AYA cancer survivors in mental tasks at work,^10^ translating into productivity loss of USD$2250 annually per AYA survivor in the United States.^12^ The three demographic characteristics that have been shown to be associated with self‐reported cognitive function in our study are consistent with reports in the literature. Similar to results reported from the LIVESTRONG survey^25^ and an adult colorectal cancer cohort,^26^ female patients were more likely than male patients to report cognitive problems. Ethnic differences in CRCI are also not unexpected given that genetic factors have been shown to be associated with varying degrees of susceptibility to self‐reported CRCI.^22^, ^23^, ^27^ Lastly, although smoking status has not been demonstrated to be linked to self‐reported CRCI, a smoking history of 10 pack years or more has been reported to be associated with poorer intellectual function among newly diagnosed head and neck cancer patients measured using objective neurocognitive tests.^28^

Interestingly, higher baseline FACT‐Cog scores were noted among patients who exhibited CRCI at subsequent follow‐up time points. This suggests that patients who eventually demonstrated significant CRCI were likely to have fewer cognitive complaints or better self‐reported cognitive function at baseline. It has previously been found (albeit among breast cancer patients) that a low, rather than high, cognitive reserve increases the risk of developing CRCI.^29^ Therefore, a more plausible explanation for our observation could be that AYA patients with high‐performing roles have a lower tendency to report cognitive issues but are more sensitive to cognitive changes due to the nature of their work and daily routine. As a result, this group of patients initially had fewer cognitive complaints but was more likely to perceive CRCI over time. Alternatively, this trend could merely be an example of regression toward the mean. Nevertheless, further investigation is warranted by comparing these observed trends against those of age‐matched noncancer controls. If higher baseline self‐reported cognitive function is indeed associated with an increased risk of CRCI in AYA cancer patients, this may imply that the risk profile of CRCI‐susceptible individuals in the AYA age range could be different and should not be indiscriminately extrapolated from findings of studies conducted among older adults.

Our study has also provided an opportunity to evaluate DT as a screening tool for CRCI. Although validated PRO measures, such as FACT‐Cog, have been widely recommended for monitoring and improving symptom management in the cancer population,^30^ the large number of questions on the FACT‐Cog questionnaire hinders its usefulness in the clinical setting. In this context, DT has the advantage of brevity. DT was designed as a brief screening tool for distress and patient concerns, including problems with tasks involving memory and concentration. The use of DT to predict subjective cognitive complaints is also supported by results from a previous study.^31^ However, in that study, overall distress, rather than reports of problems with memory and concentration, was used as the predicting factor. In our analysis, moderate agreement between the cognitive items on the DT problem checklist and PCI domain scores, which are aligned to the cognitive concerns of cancer patients, was demonstrated. This suggests that single‐item questionnaires are potentially useful to quickly screen for CRCI in a clinical setting. Nevertheless, PRO measures with established psychometric properties, such as FACT‐Cog, and neuropsychological assessments should still be utilized for a complete evaluation and monitoring of CRCI among cancer patients.

## 5 CLINICAL IMPLICATIONS

Similar to older cancer patients, AYA cancer patients experience CRCI. If left unaddressed and unmanaged, CRCI can lead to devastating economic impact on AYA cancer patients and society at large. Therefore, with improved understanding of this subject, effective strategies can be formulated to promote awareness of CRCI and mitigate its negative effects among AYA cancer patients.

## 6 STUDY LIMITATIONS

Our study had several limitations. Firstly, only 52% of participants had complete data at all time points. Low retention rates are characteristic of clinical studies involving AYA cancer patients.^32^ The data collected in our study is therefore still clinically valuable despite of the high attrition rate. Furthermore, participants with and without complete data at all time points were largely similar in demographic and clinical characteristics, including baseline cognitive function. Secondly, FACT‐Cog has mainly been used and validated in older cancer populations. Studies should ideally be carried out to validate the psychometric properties of this questionnaire, including the MCID, among AYA cancer patients. Thirdly, the patients recruited in our study were clinically heterogenous in nature. As AYA cancer patients were the population of interest, no patients were excluded on the basis of disease type or stage provided that there was no CNS involvement. Our approach was different from those of most CRCI studies that have historically focused on a specific tumor type. If the development of CRCI differs between different cancer types and treatment modalities, the heterogeneity of our study population could explain the absence of obvious longitudinal trends in cognitive decline and the lack of association between treatment factors and CRCI. While we acknowledge this shortcoming of the study, the value of characterizing CRCI specifically in the AYA population should not be understated. An important observation was made in which a proportion of AYA patients experienced CRCI over time after cancer diagnosis.

This report represents the secondary analysis of data from a longitudinal study investigating distress among AYA patients. Hence, further work is necessary to validate our findings. A prospective cohort study (ClinicalTrials.gov identifier:NCT03476070) has been launched for this purpose, incorporating recommendations from the ICCTF^1^ to improve on limitations of this study. An age‐matched noncancer control arm will be included to determine if the cognitive deterioration observed can be attributed to causes unrelated to cancer. Objective cognitive outcomes of the study participants will also be evaluated in the learning and memory, processing speed and executive function domains. This will provide further insight into specific brain areas that are affected by CRCI and illustrate whether the underlying pathology of CRCI is different in AYA cancer patients compared to other age groups.

## 7 CONCLUSIONS

Our study addressed an important and neglected research gap within CRCI research. Although patients were relatively young and without CNS disease involvement, one‐third of AYA cancer patients experienced significant decline in cognitive function. Demographic characteristics, such as gender, ethnicity, and smoking status were also found to be linked to poor self‐reported cognitive function. Nevertheless, given the exploratory nature of our analysis, future work is needed with adequately powered studies which incorporate noncancer controls and neuropsychological assessments.

## CONFLICT OF INTEREST

The authors have declared no competing interest.

## Supporting information


**Appendix** **S1.** Supporting Information.

**Appendix** **S2.** Supporting Information.

**Appendix** **S3.** Supporting Information.

**Appendix** **S4.** Supporting Information.

**Appendix** **S5.** Supporting Information.

**Appendix** **S6.** Supporting Information.

## Data Availability

The data that support the findings of this study are available from the corresponding author upon reasonable request.
