# Supplementary material for: Self‐reported cognitive outcomes among adolescent and young adult patients with noncentral nervous system cancers
Source: Psychooncology. 2020 Jul 9;29(8):1355–62. doi: 10.1002/pon.5456 (PMC7497100; doi:10.1002/pon.5456)
Supplement: Supplementary file 1 — Appendix S1. Supporting Information. [file PON-29-1355-s001.pdf]

Supporting Information 1 Distress and symptom burden of participants

1.1 Distress Thermometer (DT)

The Distress Thermometer (DT) is a brief screening tool that measures distress. Respondents are asked to indicate on a numerical scale of 0 to 10 the level of distress that they have experienced in the past week(1). A problem checklist is also provided for respondents to identify tasks or items that have posed difficulties for them in the past week. A total of 43 items, which include a single item indicating issues with memory or concentration, are available on the checklist. DT has previously been validated as a suitable tool for distress screening among cancer patients and a score of ≥5 was shown to represent clinically significant distress(2,3).

1.2 Rotterdam Symptom Checklist (RSCL)

The Rotterdam Symptom Checklist (RSCL) is used to assess the symptom burden of cancer patients. Respondents are requested to indicate the extent to which they were bothered by specific symptoms in the past week on a 4-point Likert scale. Items on the RSCL encompass four domains: (i) physical (23 items); (ii) psychological (7 items); (iii) activity levels (8 items); and (iv) overall valuation of life (1 item). Higher scores suggest greater symptom burden or impairment. In this study, RSCL was also used to measure anxiety/depressive symptoms, indicated by a score of >16 in the psychological domain(4), and significant fatigue, indicated by a score of >3 on the fatigue item(5). RSCL has been widely used and validated in the cancer population(4).

1.3 Summary of distress and symptom burden of participants

The proportion of participants reporting significant distress was highest at T1 with almost half of the study subjects (45.1%) indicating a distress score of ≥5 on DT. Psychological symptom

burden, as measured by RSCL, was also greatest at T1 while physical symptom burden peaked at T2 (1 month post-baseline). Distress and symptom burden decreased steadily after T2 and plateaued from T3 (6 months post-baseline) onwards (Table S1.1).

Table S1.1 Distress and symptom burden of participants over time

Timepoint	DT		RSCL	
	Distress level, mean (SD)†	Patients reporting significant distress, n (%)‡	Physical symptoms, mean (SD)§	Psychological symptoms, mean (SD)§
Baseline (T1) (N = 91)	4.0 (2.7)	41 (45.1)	13.9 (12.1)	28.4 (23.0)
1 month after (T2) (N = 71)	3.2 (2.7)	24 (33.8)	16.4 (16.3)	20.6 (20.0)
6 months after (T3) (N = 70)	2.7 (2.9)	22 (31.4)	10.0 (10.7)	15.5 (17.5)
12 months after (T4) (N = 58)	2.7 (2.4)	15 (25.9)	9.3 (9.8)	15.8 (17.9)

†Distress is measured on a scale from 0 to 10 with higher scores indicating greater distress.
‡Participants are considered to have significant distress with a DT score of 5 and above.
§Symptom burden is measured on a scale of 0 to 100 with higher scores indicating greater symptom burden.

1.4 References

1. National Comprehensive Cancer Network. Distress Management (Version 2.2018) [Internet]. [cited 2018 Dec 1]. Available from: https://www.nccn.org/professionals/physician_gls/pdf/distress.pdf

2. Chan A, Poon E, Goh WL, Gan Y, Tan CJ, Yeo K, et al. Assessment of psychological distress among Asian adolescents and young adults (AYA) cancer patients using the distress thermometer: a prospective, longitudinal study. Support Care Cancer [Internet]. 2018 Sep 11;26(9):3257–66. Available from: http://link.springer.com/10.1007/s00520-018-4189-y

3. Lim HA, Mahendran R, Chua J, Peh CX, Lim SE, Kua EH. The Distress Thermometer as an ultra-short screening tool: A first validation study for mixed-cancer outpatients in Singapore. Compr Psychiatry [Internet]. 2014;55(4):1055–62. Available from: http://dx.doi.org/10.1016/j.comppsych.2014.01.008

4. de Haes J, Olschewski M, Fayers P, Visser M, Cull A, Hopwood P, et al. Measuring the quality of life of cancer patients with the Rotterdam Symptom Checklist (RSCL): A manual. 2nd ed. Groningen: Northern Centre for Healthcare Research, University of Groningen; 2012. 1–39 p.

5. Smets E, Garssen B, Cull A, de Haes J. Application of the multidimensional fatigue inventory (MFI-20) in cancer patients receiving radiotherapy. Br J Cancer [Internet]. 1996 Jan 1;73(2):241–5. Available from: http://www.nature.com/articles/bjc199642
