# Supplementary figures and images for: Self‐reported cognitive outcomes among adolescent and young adult patients with noncentral nervous system cancers
Source: Psychooncology. 2020 Jul 9;29(8):1355–62. doi: 10.1002/pon.5456 (PMC7497100; doi:10.1002/pon.5456)

## Supporting Information 2 Study flow diagram

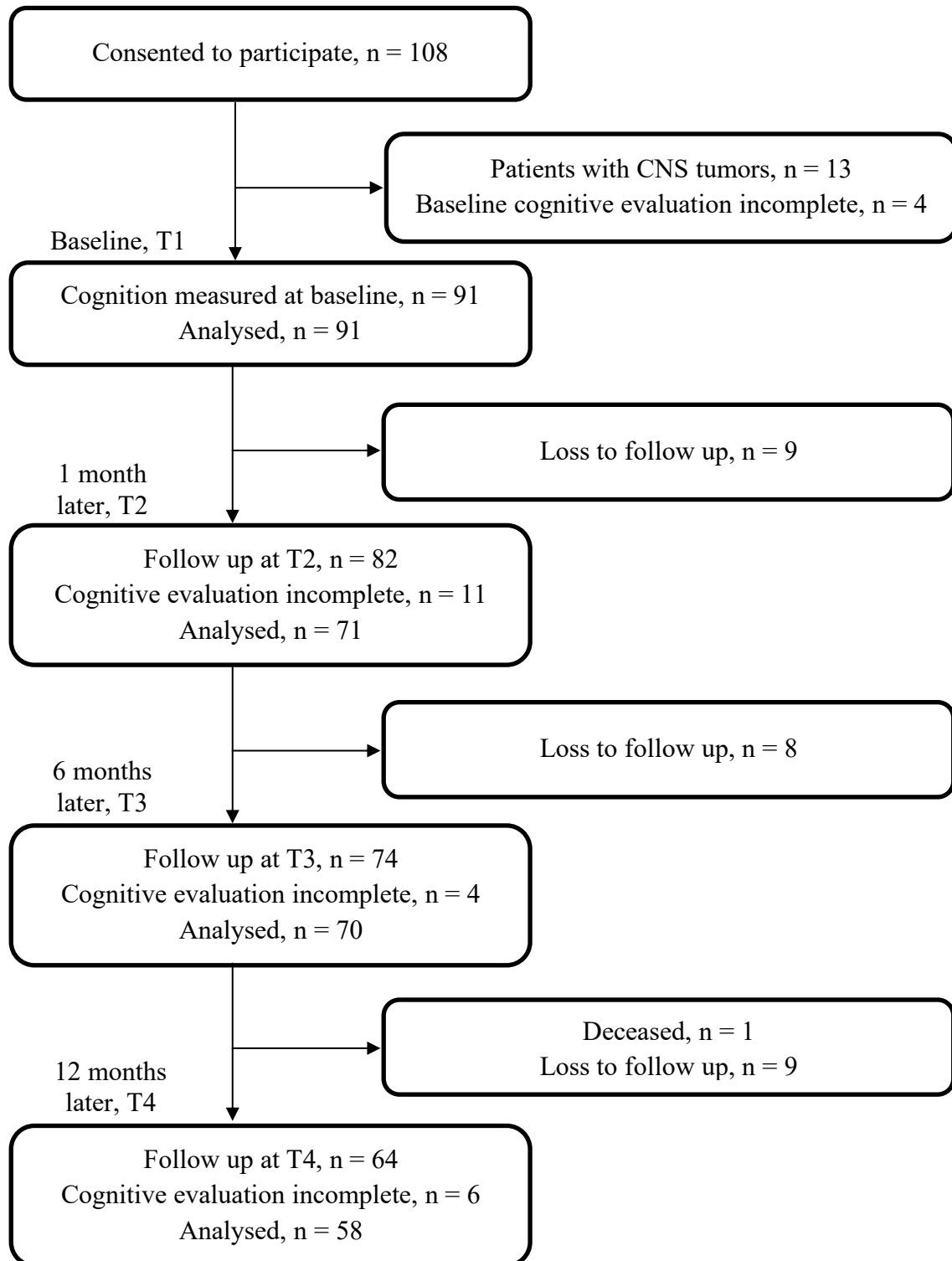

Supplement: Supplementary file 2 — Appendix S2. Supporting Information. [file PON-29-1355-s002.pdf]
