# Supplementary material for: Self‐reported cognitive outcomes among adolescent and young adult patients with noncentral nervous system cancers
Source: Psychooncology. 2020 Jul 9;29(8):1355–62. doi: 10.1002/pon.5456 (PMC7497100; doi:10.1002/pon.5456)
Supplement: Supplementary file 3 — Appendix S3. Supporting Information. [file PON-29-1355-s003.pdf]

Supporting Information 3 Comparison of characteristics between participants with complete data and those without at each time point

Characteristics	T2 (N = 91)			T3 (N = 82)			T4 (N = 74)		
	Complete data *n* = 71	Dropped out/ incomplete data n = 20	p-value	Complete data n = 70	Dropped out/ incomplete data n = 12	p-value	Complete data n = 58	Dropped out/ incomplete data n = 16	p-value
Age in years, mean (SD)	27.8 (6.7)	30.8 (6.2)	0.08	28.2 (6.6)	29.5 (8.0)	0.54	28.4 (6.7)	28.3 (7.2)	0.93
Time after diagnosis in months, median (IQR)	0.9 (0.2- 1.7)	0 (0-14.1)	0.14	0.7(0-1.7)	1.0 (0.8-2.4)	0.18	0.7 (0-1.7)	0.8 (0-1.6)	0.99
Gender, n (%)			0.37			0.37			0.35
Male	40 (56.3)	9 (45.0)		37 (52.9)	8 (66.7)		33 (56.9)	7 (43.8)	
Female	31 (43.7)	11 (55.0)		33 (47.1)	4 (33.3)		25 (43.1)	9 (56.2)	
Race, n (%)			0.11			0.37			0.32
Chinese	53 (74.6)	11 (55.0)		51 (72.9)	8 (66.7)		46 (79.3)	9 (56.2)	
Malay	7 (9.9)	1 (5.0)		8 (11.4)	0 (0.0)		5 (8.6)	3 (18.8)	
Indian	3 (4.2)	3 (15.0)		3 (4.3)	1 (8.3)		2 (3.4)	1 (6.2)	
Others[†]	8 (11.3)	5 (25.0)		8 (11.4)	3 (25.0)		5 (8.6)	3 (18.8)	
Highest education level, n (%)			0.35			0.75			0.48
Primary/Secondary education	8 (11.3)	2 (10.0)		7 (10.0)	1 (8.3)		5 (8.6)	3 (18.8)	
Pre-university	18 (25.4)	3 (15.0)		19 (27.1)	2 (16.7)		16 (27.6)	3 (18.8)	
Undergraduate	25 (35.2)	4 (20.0)		24 (34.3)	4 (33.3)		19 (32.8)	7 (43.8)	
Postgraduate	20 (28.2)	9 (45.0)		19 (27.1)	5 (41.7)		17 (29.3)	3 (18.8)	
Unreported	0 (0.0)	2 (10.0)		1 (1.4)	0 (0.0)		1 (1.7)	0 (0.0)	
Smoking status, n (%)			0.58			0.67			0.44
No history of smoking	49 (69.0)	13 (65.0)		46 (65.7)	9 (75.0)		39 (67.2)	9 (56.2)	
Currently smoking	11 (15.5)	1 (5.0)		10 (14.3)	2 (16.7)		7 (12.1)	4 (25.0)	
Previously smoking	11 (15.5)	3 (15.0)		13 (18.6)	1 (8.3)		11 (19.0)	3 (18.8)	
Unreported	0 (0.0)	3 (15.0)		1 (1.4)	0 (0.0)		1 (1.7)	0 (0.0)	

Characteristics	T2 (N = 91)			T3 (N = 82)			T4 (N = 74)		
	Complete data *n* = 71	Dropped out/ incomplete data n = 20	p-value	Complete data n = 70	Dropped out/ incomplete data n = 12	p-value	Complete data n = 58	Dropped out/ incomplete data n = 16	p-value
Alcohol, n (%)			0.11			0.18			0.91
No	23 (32.4)	9 (45.0)		21 (30.0)	6 (50.0)		17 (29.3)	5 (31.2)	
Yes	48 (67.6)	8 (40.0)		48 (68.6)	6 (50.0)		40 (69.0)	11 (68.8)	
Unreported	0 (0.0)	3 (15.0)		1 (1.4)	0 (0.0)		1 (1.7)	0 (0.0)	
Cancer type, n (%)			0.008			0.74			0.65
Sarcoma	28 (39.4)	2 (10.0)		24 (34.3)	5 (41.7)		21 (36.2)	5 (31.2)	
Lymphoma	25 (35.2)	14 (70.0)		26 (37.1)	5 (41.7)		23 (39.7)	5 (31.2)	
Germ cell tumor	12 (16.9)	0 (0.0)		10 (14.3)	2 (16.7)		8 (13.8)	2 (12.5)	
Melanoma	5 (7.0)	3 (15.0)		8 (11.4)	0 (0.0)		5 (8.6)	3 (18.8)	
Pancreatic neoplasm	1 (1.4)	1 (5.0)		2 (2.9)	0 (0.0)		1 (1.7)	1 (6.2)	
Cancer stage, n (%)			0.97			0.29			0.66
I	21 (29.6)	6 (30.0)		22 (31.4)	2 (16.7)		20 (34.5)	2 (12.5)	
II	14 (19.7)	4 (20.0)		16 (22.9)	1 (8.3)		13 (22.4)	3 (18.8)	
III	9 (12.7)	2 (10.0)		9 (12.9)	2 (16.7)		8 (13.8)	2 (12.5)	
IV	19 (26.8)	4 (20.0)		15 (21.4)	5 (41.7)		13 (22.4)	4 (25.0)	
Not applicable	8 (11.3)	4 (20.0)		8 (11.4)	2 (16.7)		4 (6.9)	5 (31.2)	
Disease nature			0.02			0.86			0.62
New diagnosis	64 (90.1)	14 (70.0)		63 (90.0)	11 (91.7)		52 (89.7)	15 (93.8)	
Relapsed/refractory disease	7 (9.9)	6 (30.0)		7 (10.0)	1 (8.3)		6 (10.3)	1 (6.2)	
Treatment modality									
Surgery	33 (46.5)	11 (55.0)	0.50	35 (50.0)	5 (41.7)	0.59	28 (48.3)	7 (43.8)	0.75
Chemotherapy	49 (69.0)	3 (15.0)	<0.001	45 (64.3)	7 (58.3)	0.69	38 (65.5)	9 (56.2)	0.50
Radiotherapy	22 (31.0)	3 (15.0)	0.16	22 (31.4)	2 (16.7)	0.30	18 (31.0)	4 (25.0)	0.64
Baseline FACT-Cog, mean (SD)[‡]	126.4 (22.3)	119.4 (36.4)	0.29	126.4 (23.0)	127.6 (19.1)	0.86	127.4 (21.7)	124.5 (25.7)	0.65

[†]Burmese, Filipino and Arabian.
