# Supplementary material for: Self‐reported cognitive outcomes among adolescent and young adult patients with noncentral nervous system cancers
Source: Psychooncology. 2020 Jul 9;29(8):1355–62. doi: 10.1002/pon.5456 (PMC7497100; doi:10.1002/pon.5456)
Supplement: Supplementary file 4 — Appendix S4. Supporting Information. [file PON-29-1355-s004.pdf]

Supporting Information 4 Concordance of cognitive outcomes between DT and FACT-Cog

Duration after baseline	DT, n (%)†	FACT-Cog, n (%)‡		% agreement	Cohen's Kappa
1 month (T2) (*N*=71)	12 (16.9)	Total score	16 (22.5%)	74.7%	0.20
		PCI domain score	10 (14.1%)	88.7%	0.57
6 months (T3) (*N*=70)	10 (14.3)	Total score	18 (25.7%)	74.3%	0.21
		PCI domain score	7 (10.0%)	92.9%	0.67
12 months (T4) (*N*=58)	11 (19.0)	Total score	12 (20.7%)	74.1%	0.19
		PCI domain score	8 (13.8%)	84.5%	0.44

†Patients indicating memory and concentration as a concern on the DT problem list

‡Patients experiencing CRCI based on MCID established for FACT-Cog total score and cut-off established for PCI subscale score.
