# Supplementary material for: Self‐reported cognitive outcomes among adolescent and young adult patients with noncentral nervous system cancers
Source: Psychooncology. 2020 Jul 9;29(8):1355–62. doi: 10.1002/pon.5456 (PMC7497100; doi:10.1002/pon.5456)
Supplement: Supplementary file 5 — Appendix S5. Supporting Information. [file PON-29-1355-s005.pdf]

**Supporting Information 5 Model diagnostics for mixed model analysis**

| Model | AIC† | BIC† |
|---|---|---|
| Inclusion of variables of interest (treatment modalities) | | |
| Model 1: Chemotherapy + Surgery + Radiotherapy + Timepoint | 2560 | 2593 |
| Inclusion of demographic variables | | |
| Model 2: Model 1 + Age + Gender | 2561 | 2601 |
| Model 3: Model 2 + Ethnicity | 2545 | 2596 |
| Model 4: Model 2 + Education level | 2522 | 2573 |
| Model 5: Model 2 + Ethnicity + Education level | 2508 | 2571 |
| Inclusion of psychosocial variables | | |
| Model 6: Model 1 + Anxiety/Depressive symptoms + Fatigue | 2485 | 2526 |
| Model 7: Model 2 + Anxiety/Depressive symptoms + Fatigue | 2486 | 2534 |
| Model 8: Model 5 + Anxiety/Depressive symptoms + Fatigue | 2437 | 2507 |
| Inclusion of lifestyle habits | | |
| Model 9: Model 8 + Smoking | 2423 | 2499 |
| Model 10: Model 8 + Alcohol consumption | 2430 | 2503 |
| Model 11: Model 8 + Smoking + Alcohol consumption | 2424 | 2504 |
| After stepwise removal of variables | | |
| Final model: Model 9 – Age – Education level (Model 1 + Gender + Ethnicity + Anxiety/Depressive symptoms + Fatigue + Smoking) | 2418 | 2480 |

† AIC and BIC are conventionally used in mixed-effects models to guide model building, especially when non-nested models are compared. Lower AIC and BIC values indicate model stability
