# Supplementary material for: Self‐reported cognitive outcomes among adolescent and young adult patients with noncentral nervous system cancers
Source: Psychooncology. 2020 Jul 9;29(8):1355–62. doi: 10.1002/pon.5456 (PMC7497100; doi:10.1002/pon.5456)
Supplement: Supplementary file 6 — Appendix S6. Supporting Information. [file PON-29-1355-s006.pdf]

Supporting Information 6 Factors associated with cognitive function (measured based on PCI subscale score) among AYA cancer patients (n=91)

	Beta coefficient	95% CI	p-value
Chemotherapy			0.50
No	Reference		
Yes	1.29	–2.4 to 5.0	
Surgery			0.79
No	Reference		
Yes	–0.53	–4.3 to 3.3	
Radiotherapy			0.11
No	Reference		
Yes	–3.5	–7.6 to 0.7	
Gender			<0.01
Male	Reference		
Female	–5.4	–9.0 to –1.7	
Ethnicity			<0.01
Chinese	Reference		
Malay	1.7	–5.2 to 8.6	
Indian	–19.5	–27.1 to –12.0	
Others†	2.4	–2.9 to 7.8	
Anxiety/depressive symptoms‡			<0.01
No	Reference		
Yes	–9.7	–13.4 to –6.1	
Fatigue§			<0.01
No	Reference		
Yes	–4.0	–6.5 to –1.5	
Smoking habit			0.02
No history of smoking	Reference		
Currently smoking	–8.8	–14.8 to –2.7	
Previously smoking	–2.9	–8.0 to 2.2	

†Burmese, Filipino and Arabian

‡RSCL psychological scale score >16

§RSCL fatigue item score >2
